# Long-Term Efficacy and Safety of755-nm Alexandrite Laser for Axillary Hair Removal: ِA Comparative Analysis of Single vs. DualFlash lamp Systems

**DOI:** 10.2196/76523

**Published:** 2025-11-21

**Authors:** Kawthar Shurrab, Manal Asad Nassr

**Affiliations:** 1 Biomedical Photonics Lab, Higher Institute for Laser Research and Applications, Damascus University, Airport Highway, Damascus, 96311, Syrian Arab Republic, 963 988911577

**Keywords:** hair removal, 755-nm laser, single flash lamp, dual flash lamp, photothermolysis, axillary hair reduction, alexandrite laser

## Abstract

**Background:**

Laser hair removal is a noninvasive cosmetic procedure that targets melanin in hair follicles through selective photothermolysis.

**Objective:**

This study aims to evaluate the long-term efficacy and safety of the 755-nm alexandrite laser for axillary hair removal by comparing single- and dual-flash lamp systems.

**Methods:**

In total, 40 women aged 20 to 35 years with Fitzpatrick skin types II and III participated in a study on laser hair removal for their axillae. Participants underwent 3 treatment sessions, each spaced 4 weeks apart, from January to April 2024. They were divided into 2 groups, both receiving treatments with an alexandrite laser (755 nm, 14 mm spot size). The first group was treated with a dose of 8 J/cm² using a single-flash lamp device operating at 5 Hz, with a pulse duration of 10 ms. The second group received doses between 9 and 11 J/cm² from a dual-flash lamp device operating at 2.5 Hz, with pulse durations ranging from 10 to 15 ms. Photographs and hair counts were taken at baseline and 1 month after the final session. A 2-tailed *t* test was used to assess statistical significance, and regression analysis evaluated treatment effects. Pain scores and side effects were assessed using a visual analog scale in a satisfaction questionnaire.

**Results:**

The dual-flash lamp laser achieved an overall hair reduction of 94%, while the single-flash lamp laser resulted in a 91% reduction in the axilla. The difference was not statistically significant (*P*=.14). No serious adverse effects were reported with either device, indicating effective safety features.

**Conclusions:**

The outcomes show that both systems provide similar results in terms of efficacy and safety, with no reported side effects, and results were maintained even after 6 and 12 months of follow-up.

## Introduction

Laser hair removal (LHR) is a widely used and effective approach for long-term hair reduction [1,2]. The technique is grounded in the principle of selective photothermolysis, wherein laser pulses target melanin in hair follicles to induce photothermal destruction while preserving surrounding tissue [3-5]. Wavelengths between 600 and 1100 nm are particularly effective for this purpose, as they efficiently absorb melanin, ensuring effective follicular stem cell disruption. Common devices for LHR include long-pulsed lasers (eg, ruby, alexandrite, diode, and neodymium-doped yttrium aluminum garnet) and intense pulsed light systems [6-9]. Among these, the 755-nm alexandrite laser is considered the gold standard due to its superior melanin absorption and efficacy, particularly for skin types I to III [10-12]. Its long pulses maximize follicle energy absorption while reducing skin damage risk [13-15].

Lasers operate through flashlamp pumping, which uses gas-filled tubes, typically xenon or krypton, to generate high-intensity light that excites the laser medium [16,17]. This technology has significantly advanced LHR devices. Many systems use a single flashlamp to target hair follicles while protecting surrounding tissues, offering reliability and low maintenance. In contrast, dual-flashlamp–pumped systems, which feature 2 flashlamps adjacent to the laser crystal, provide uniform pumping and enhanced stability, enabling rapid pulses and higher energy fluence [18]. This enhances treatment efficiency and reduces session durations, making it ideal for larger areas. The effectiveness of alexandrite systems depends on their energy delivery, while single-flashlamp devices use low fluence with higher repetition rates, allowing gradual thermal accumulation in hair follicles. Dual-flashlamp configurations offer higher fluence and more uniform energy output, enhancing treatment efficiency for larger areas. Recent research indicates that treatment outcomes are influenced more by the energy delivery method than by absolute energy levels, affecting follicular thermal thresholds and hair reduction durability [19,20]. In addition to hair removal, alexandrite lasers have shown promising applications in other dermatologic contexts. A recent split-body clinical trial demonstrated the efficacy and safety of these systems in treating keratosis pilaris, confirming that optimized fluence and pulse duration settings can extend their therapeutic applications beyond traditional photoepilation [15] to include vascular and pigmented lesion treatments [21]. Moreover, novel approaches in LHR emphasize expanding safety profiles across all skin types, highlighting the role of advanced epidermal cooling and energy modulation in minimizing side effects [22]. Although emerging platforms combining multiple wavelengths (810, 940, and 1060 nm) are increasingly adopted for treating fine or less pigmented hairs, the alexandrite laser remains the benchmark for comparative evaluation, offering clinicians robust evidence to optimize treatment protocols [23]. This study represents the first direct comparison between single- and dual-flashlamp alexandrite laser systems for hair removal using the 755-nm wavelength. Unlike previous research that focused solely on single-flash devices and short-term outcomes, our work provides a detailed analysis of treatment parameters, fluence, frequency, pulse duration, and the number of passes per session and includes a comprehensive 12-month follow-up, which enhances the reproducibility and clinical utility of our findings. By integrating patient-centered considerations such as session cost, treatment duration, and side effect profiles, this study offers novel insights that support personalized decision-making in clinical dermatology and contribute to optimizing long-term hair removal protocols.

## Methods

### Ethical Considerations

The comparative study methods and protocols were approved by the Ethics Committee of Damascus University (approval ID: HILRA-261124-372). All participants voluntarily took part in the study and provided written informed consent prior to enrollment. No financial or material compensation was provided, and participants did not receive any free treatments or benefits in exchange for participation. Before enrollment, all participants were informed about the complete treatment protocol and provided written consent for data collection and the publication of their images. They were also informed that participation was entirely voluntary and that they could withdraw at any time without penalty. All identifying information was kept confidential, with only anonymized reference numbers used throughout data collection, storage, and analysis.

### Clinical Data

The study was conducted at a private laser clinic in Damascus, Syria, from January to April 2024. The participants in the study were aged between 20 and 35 years, with a mean age of 31.3 (SD 4.3) years for one group and mean age of 30.1 (SD 4.2) years for another. None of the participants had previously undergone laser treatments in the axillary area, and they were classified as Fitzpatrick skin types II and III. All participants had dark terminal hairs. We screened interested female participants with unwanted axillary hair who were first-time laser users and met the inclusion criteria for participation. Exclusion criteria included individuals who were suntanned, those with contraindications to laser treatment, and those who were not willing or committed to following the required precare and postcare procedures. Female participants were chosen for the study to focus on one sex and to minimize variables such as hormonal differences between sexes [24]. The study excluded individuals who did not meet certain eligibility criteria, including those with blonde, red, or light-colored hair in the axillary region; those with tanned or sun-exposed skin; and those who were pregnant or breastfeeding. Additional exclusions are applied to individuals with a history of seizures, prior laser treatments in the underarm area, skin infections, or those prone to hypertrophic scarring or keloids.

After establishing the study sample, each participant was assigned a randomly generated reference number to allocate them to a specific laser machine [25].

Participants on medications such as isotretinoin, antibiotics, or anticoagulants, as well as those with tattoos in the treatment area, joint pain during gold therapy, contagious diseases, or diabetes, were also disqualified. Other criteria included those with suspicious pigmented lesions, users of photosensitive medications, and patients undergoing radiation or chemotherapy.

### Research Design

All 40 participants were randomly assigned to 2 groups, A and B, simultaneously. A number generator was used to ensure that the allocation to the treatment groups was concealed, thereby reducing bias and enhancing the reliability of the results [25,26]. Participants were assigned to receive hair removal treatments using either the DEKA Motus Axe 755-nm alexandrite laser (DEKA Laser), which features a 20 mm spot size and uses single-flashlamp technology, along with a Moveo handpiece that allows for continuous motion delivery instead of traditional single pulses and provides epidermal cooling, or the DEKA Again 755-nm alexandrite laser (DEKA Laser). The latter also has a 20 mm spot size and uses dual-flashlamp technology combined with air cooling down to −20 °C, minimizing pain and thermal damage.

Participants in the study received 3 treatment sessions, scheduled 4 weeks apart. No topical anesthetics or medications were used during these sessions, and patients reported no discomfort or redness. They were instructed to avoid all other methods of hair removal, except for shaving. They were allowed to shave the area 2 weeks after the laser session if needed.

Each participant received aftercare and treatment instructions, including guidelines for shaving to ensure uniformity. They were asked to shave their underarm hair with a razor 3 days before their treatment. Additionally, they were advised to use a broad-spectrum sunscreen and to avoid heat, humidity, sweating, friction, rubbing, cosmetics, and salon procedures for 3 to 5 days after each laser session.

A single technician conducted all sessions. After completing the 3 sessions, treatment was paused for evaluation. Posttreatment evaluations were performed at 3 time points: 1 month after the final session, 6 months thereafter, and at the 12-month follow-up.

The participants were divided into 2 groups: group A received 755-nm alexandrite treatments using the DEKA Motus AX device, with a dose of 8 J/cm², a frequency of 5 Hz, and a pulse duration of 10 milliseconds, and group B underwent 755-nm alexandrite treatments using the DEKA Again device, with a dose ranging from 9 to 11 J/cm², a frequency of 2.5 Hz, and a pulse duration between 10 and 15 milliseconds. The entire area was treated by completing 1 pass horizontally per session.

### Evaluation and Outcome Measures

Clinical photographs were taken before the first treatment session, 1 month after the last session, and at a final follow-up appointment scheduled 12 months after treatment. This was done using a mobile device to assess hair counts and thickness [27-29]. Hair counts were analyzed using HowMany AI (YesChat AI), a free object-counting software tool whose accuracy was validated by comparison with manual counting [30].

Patients were instructed to measure their pain intensity during the LHR sessions using a 10-cm Visual Analog Scale. Pain levels were systematically classified according to the following criteria: no pain (0), mild pain (1-3), moderate pain (4-6), and severe pain (7-10) [7-10,31,32].

After completing the treatment, patients received a questionnaire to evaluate their satisfaction with the level of improvement or any adverse effects. The ratings were categorized into 4 groups: mild (<25% improvement), moderate (25% to <50% improvement), good (50% to <75% improvement), and very good (>75% improvement) [33].

### Data Analysis

All collected data were recorded and analyzed using the SPSS software (version 26.0; IBM Corp). Various descriptive and inferential statistical techniques were used. Statistical descriptors, such as tables, means, and percentages, were used to analyze patterns within the collected data. Additionally, Microsoft Excel was used to enhance visual representation through the generation of relevant charts and graphs.

During statistical analysis, data were assessed using either parametric or nonparametric tests to determine the normality of the variables based on their distribution. A *t* test was used to compare differences within groups, while an analysis of covariance (ANCOVA) regression analysis was used to determine the impact of these differences. A *P* value <.05 was considered statistically significant. To evaluate the differences between the 2 technologies, we compared the percentage of hair reduction achieved with the dual-flashlamp technology to that obtained with the single-flashlamp technology, using the following equation [34]:


$$\Delta \text{Reduction}=\left[\frac{H_{b,\text{dual}}-H_{p,\text{dual}}}{H_{b,\text{dual}}}-\frac{H_{b,\text{single}}-H_{p,\text{single}}}{H_{b,\text{single}}}\right]\times 100$$

Where *H*_*b,*dual_ and *H*_*p*,dual_ represent the baseline and posttreatment hair counts for the dual-flashlamp group, and *H*_*b,*single_ and *H*_*p*,single_ represent the baseline and posttreatment counts for the single-flashlamp group.

## Results

### Assessment Scores

A total of 40 Syrian female patients, aged between 20 and 35 years (with a mean age of 31.3, SD 4.3, years in one group and 30.1, SD 4.2, years in another), participated in this study. According to the Fitzpatrick skin type classification, 17 (43%) patients were classified as skin phototype II, while 23 (58%) patients were classified as skin phototype III.

For the treatment, the initial fluence parameters were set as follows: for the single-flashlamp laser group, the settings were 8 J/cm², a frequency of 5 Hz, and a pulse duration of 10 milliseconds. For the dual-flashlamp laser group, the parameters ranged from 9 to 11 J/cm², with a frequency of 2.5 Hz and a pulse duration between 10 and 15 milliseconds.

The findings indicated significant hair reduction in both groups when comparing baseline hair counts to measurements taken 1 month after the final 3 treatment sessions. Statistically significant differences were observed in both groups (*P*< .001), highlighting the effectiveness of the treatment.

**Figure 1.**
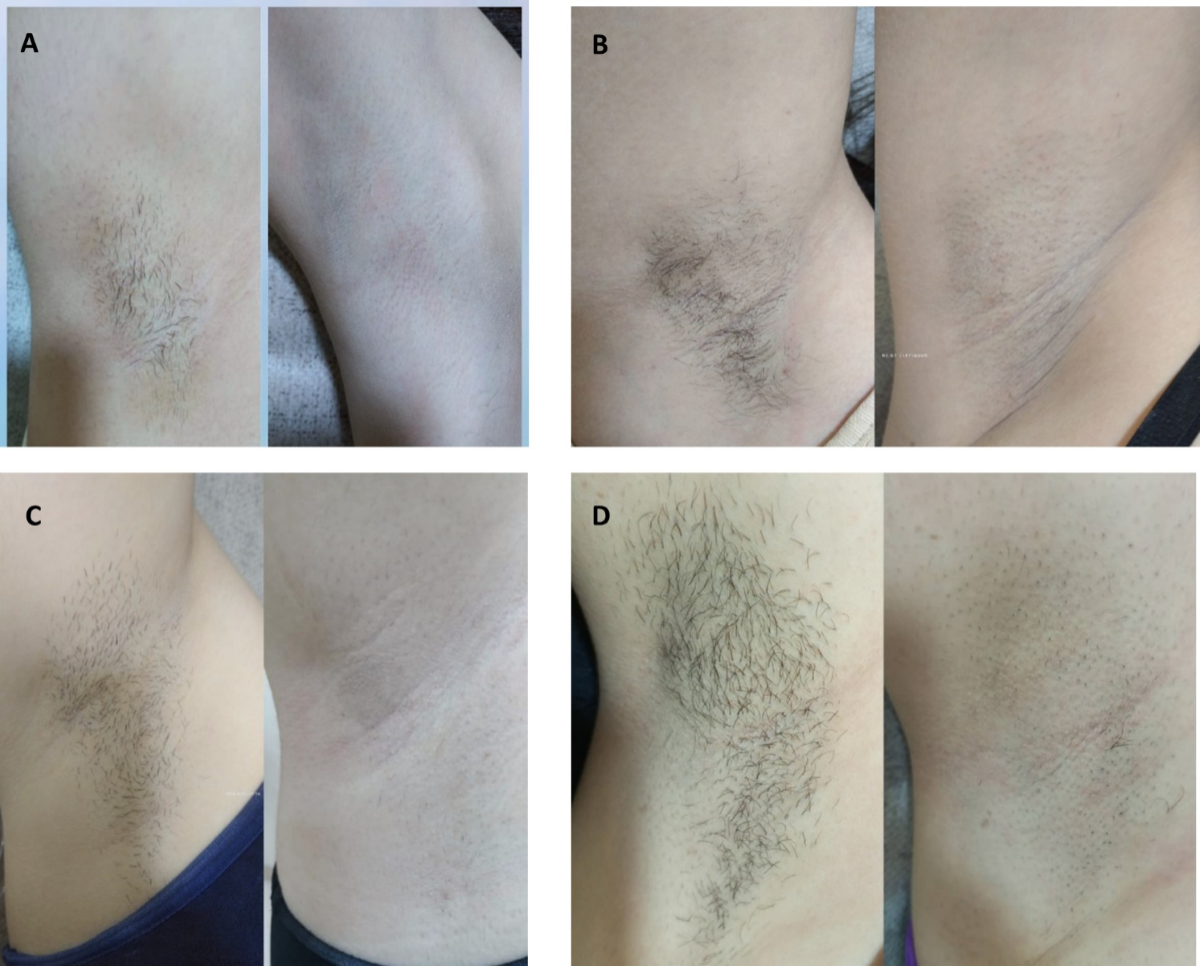
Clinical photographs taken at baseline and 1 month after the final session of 3 treatments show a significant decrease in hair count. (A) A 26-year-old female patient with skin phototype II and (B) a 31-year-old female patient with skin phototype III from group A were treated with single-flashlamp technology, while (C) a 27-year-old female patient with skin phototype II and (D) a 30-year-old female patient with skin phototype III from group B were treated with dual-flashlamp technology.

Visual documentation in Figure 1 and Table 1 supports the quantitative findings by showcasing representative baseline and posttreatment photographs of 4 patients from both groups, illustrating consistent axillary hair reduction across both skin phototypes and treatment modalities.

**Table 1.** Baseline hair counts, mean reduction, and 95% CIs after 3 treatment sessions in single- and dual-flashlamp laser systems.

	Baseline, mean (SD)	After 3 treatments, mean (SD)	Mean reduction (95% CI)	Reduction^a^ (95% CI)
Group A	293.7 (5.9)	17.6 (2.2)	268.5 (262.8 to 274.2)	91.4 (88.8 to 99.5)
Group B	297.7 (4.9)	16.6 (3.7)	279.2 (276.5 to 281.9)	94 (91.9 to 97.0)
Between-group difference	N/A^b^	N/A	10.7 (4.5 to 16.9)	2.6 (–0.5 to 5.7)

a The values are presented as percentages.

b N/A: not applicable; because the between-group comparison reflects a mean difference with a 95% CI rather than raw measurements required for mean (SD) values.

**Figure 2.**
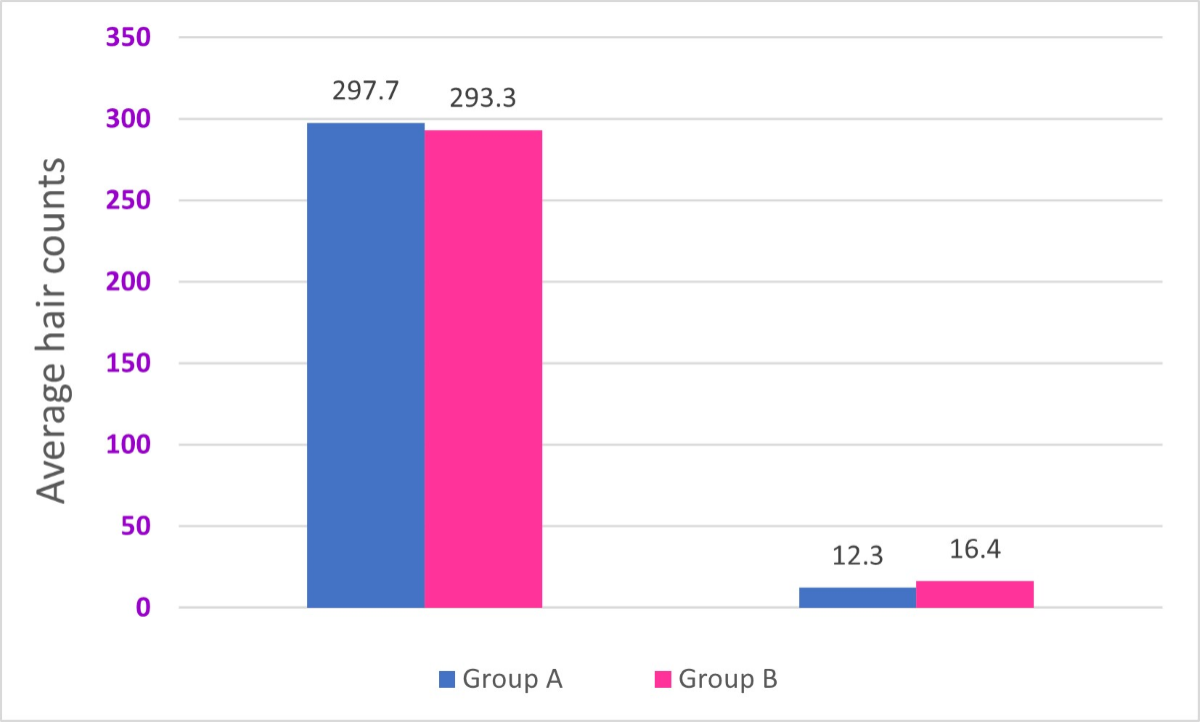
Comparative analysis of groups A and B using an independent samples test (91.4% vs 94%, with a difference of 2.7%).

The comparison of the 2 groups showed a significant reduction in hair counts after treatment as shown in Figure 2. In group A, the average hair count dropped from 293.7 (SD 5.9) at baseline to 17.3 (SD 2.2) after 3 treatments (*t*_19_=209.2; *P*<.001), yielding a mean reduction of 268.47 (95% CI 262.76-274.18) hairs, equivalent to 91.4% (95% CI 88.8%-99.5%) reduction. Similarly, group B showed a decrease from 297.7 (SD 4.9) at baseline to 16.6 (3.7) after 3 treatments (*t*_19_=107.5; *P*<.001), with a mean reduction of 279.20 (95% CI 276.49-281.91) hairs, equivalent to 94% (95% CI 91.9%-97.0%) reduction.

The between-group difference in mean reduction was 10.73 (95% CI 4.54-16.92) hairs, favoring the dual flashlamp. However, the posttreatment mean difference in residual hair count was −0.65 (95% CI −2.6 to 1.3) hairs, indicating that both technologies achieved highly comparable and clinically significant efficacy, with no statistically or clinically meaningful difference. These results align with the ANCOVA model, which confirmed no significant group effect after baseline adjustment (*P*=.14).

To evaluate the differences between the 2 laser treatments, an ANCOVA test was performed, and the results are as follows.

The ANCOVA showed no statistically significant differences between the groups (single- vs dual-flashlamp technology) for the posttreatment hair count after adjusting for baseline measurements (*F*_1,37_=2.23; *P*=.14; η²=0.06). Additionally, the baseline measurement did not significantly impact the outcome (*F*_1,37_=0.718; *P*=.40; η²=0.02). The corrected model accounted for 9% of the variance in posttreatment outcomes (*R*²=0.09), with an adjusted *R*² value of 0.04, as presented in Table 2.

**Table 2.** The analysis of covariance results comparing the single- and dual-flashlamp laser groups after 3 treatments.

Source	Type III sum of squares (*df*)		Mean square	*F* test *(df)*	*P* value	Partial η²
Corrected model^a^	15.04 (2)		7.52	1.84 (2.37)	.17	0.09
Intercept	12.99 (1)		12.99	3.17 (1.37)	.08	0.08
Baseline (before)	2.94 (1)		2.94	0.72 (1.37)	.40	0.02
Group (A vs B)	9.14 (1)		9.14	2.24 (1.37)	.14	0.06
Error	151.36 (37)		4.09	—^b^	—	—
Total	11,322.00 (40)		—	—	—	—
Corrected total	166.40 (39)		—	—	—	—

a Model fit: *R*2=0.09 (adjusted *R*2=0.04).

b Not available.

### Side Effects and Patient Satisfaction

Mild side effects were reported during the initial treatment session in both groups, but these effects diminished in subsequent sessions. Approximately 65% (13/20) of participants who received a single-flashlamp laser treatment reported experiencing pain rated as mild to moderate, and 25% (5/20) noticed slight redness or mild erythema. In contrast, 70% (14/20) of participants who underwent dual-flashlamp laser treatment reported similar pain levels, while 20% (4/20) experienced redness that only appeared during the treatment (Table 3). All these side effects disappeared within minutes after treatment. Axillary hyperhidrosis was observed following LHR in both groups. In group A, 30% (6/20) of patients reported this condition, whereas group B exhibited a higher incidence at 60% (12/20). While some participants regained normal perspiration spontaneously, others continued to experience moderate hyperhidrosis for up to 12 months. The effects were moderate for all participants who received treatment. There were no unexpected or severe adverse events recorded during any of the follow-up periods at 3, 6, and 12 months after the last treatment. Additionally, no cases of burns, hyperpigmentation, depigmentation, or paradoxical hair growth were reported in either group.

**Table 3.** Comparison of side effects between single- and dual-flashlamp groups with statistical analysis

Side effect	Single-flashlamp laser (group A), n (%)	Dual-flashlamp laser (group B), n (%)	Chi-square (*df*)	*P* value^a^	95% CI for difference
Pain (mild- to moderate)	13 (65)	14 (70)	0.1 (1)	.74	−28.2 to 38.2
Redness or erythema	5 (25)	4 (20)	0.1 (1)	.72	−23.9 to 33.9
Axillary hyperhidrosis	6 (30)	12 (60)	3.3 (1)	.07	−2.5 to 62.5

a *P* value corresponds to the Fisher exact test.

Despite the specific guidelines about shaving frequency, none of the participants in either group needed to shave between sessions.

To assess the reliability of observed differences in side effects, 95% CIs were calculated. For pain and erythema, the 95% CIs were −28.2% to 38.2% and −23.9% to 33.9%, respectively. Both intervals included 0, indicating no statistically significant differences between the 2 laser modalities in terms of tolerability. However, the 95% CI for axillary hyperhidrosis (−2.5% to 62.5%) was notably wide, suggesting a potential trend toward increased incidence in the dual-flashlamp group. Although this finding did not reach statistical significance (*P*=.07), it warrants further investigation in larger cohorts to determine whether higher-energy delivery systems contribute to this side effect.

Patient satisfaction was high for both groups. Of the total 40 patients, 6 (15%) reported their improvement as good, while the remaining 34 (85%) rated it as very good. After just 3 sessions, all participants experienced a significant reduction in hair counts, and these results remained consistent for 12 months.

## Discussion

### Principal Findings

This study presents the first direct comparison of single- and dual-flashlamp alexandrite laser systems for axillary hair removal, demonstrating substantial and sustained hair reduction in both groups, with no statistically significant difference in efficacy or safety.

Hair removal is a widely practiced cosmetic procedure among men and women, with a notably higher demand among women. Motivations for this practice include esthetic enhancement, personal comfort, and hygiene considerations. LHR has emerged as a preferred method due to its efficacy in achieving smooth skin, reducing ingrown hairs, and enhancing self-confidence [33].

Despite extensive research on laser technologies using various wavelengths [5,28,35-37], limited studies have examined the comparative efficacy of different laser systems.

Both modalities demonstrated substantial and sustained hair reduction across Fitzpatrick skin types II and III, with results maintained at 6- and 12-month follow-ups. Group A (single-flash) achieved a 91.4% (95% CI 88.8%-99.5%) reduction, while group B (dual-flash) achieved 94% (95% CI 91.9%-97.0%) reduction. Although the dual-flash system yielded a slightly higher mean reduction (10.7, 95% CI 4.54-16.9 hairs), the adjusted ANCOVA analysis confirmed no statistically significant difference (*P*=.14), reinforcing the clinical equivalence of both techniques.

The results suggest that LHR efficacy is primarily determined by energy and energy density delivery to the tissue rather than energy output or the number of flashlamps used. Low fluence, high repetition rate lasers typically found in single-flashlamp systems deliver energy in multiple passes, facilitating gradual thermal accumulation within hair follicles [19,20]. Conversely, although the dual-flashlamp technique generates higher overall energy output, hair follicles may reach their thermal damage threshold using a single-flashlamp laser, rendering additional energy ineffective in enhancing treatment outcomes. Thus, both methods produce comparable levels of follicular damage, yielding similar hair reduction results.

Importantly, our outcomes enhance reproducibility and clinical applicability of the results by detailing all treatment parameters, fluence, frequency, pulse duration, and number of passes and by incorporating a rigorous 12-month follow-up. Unlike many previous reports that lacked consistent technical documentation, our findings establish evidence-based guidance for optimizing treatment protocols [6^6^].

Patients undergoing LHR may experience a range of side effects, from mild discomfort to more severe complications [36]. Arsiwala and Majid [37] highlighted that laser treatment efficacy is influenced by both the laser device and the expertise of the practitioner. Serious adverse effects can occur when procedures are performed by untrained individuals.

Regarding safety, both systems exhibited favorable profiles. Mild to moderate pain was reported by 65% (13/20) of group A and 70% (14/20) of group B participants, with transient erythema observed in 25% (5/20) and 20% (4/20) , respectively. These side effects resolved within minutes, likely due to adherence to professional laser protocols, including optimized treatment parameters and adequate epidermal cooling before, during, and after the procedure, and were not statistically significant (pain 95% CI −28.2% to 38.2%; erythema 95% CI −23.9% to 33.9%). However, axillary hyperhidrosis was more prevalent in group B (12/20, 60%) than in group A (6/20, 30%), with a wide 95% CI −2.5% to 62.5%, suggesting a potential trend that warrants further investigation. No cases of burns, pigmentary changes, or paradoxical hair growth were reported.

Patient satisfaction was uniformly high, with 85% (34/40) rating their improvement as very good and 15% (6/40) as good. Notably, none of the participants required shaving between sessions, indicating robust follicular suppression.

From a practical standpoint, the comparable efficacy of both systems allows clinicians to prioritize other factors in device selection. Single-flash systems may offer cost advantages and slightly reduced side effects, while dual-flash systems may provide faster treatment times and enhanced device longevity. These considerations are especially relevant in resource-limited settings or high-throughput clinics.

### Limitations and Strengths

Several limitations need to be acknowledged. First, the relatively small sample size (n=40) may have limited the statistical power to detect minor differences between the 2 treatment modalities. Second, the inclusion of only female participants restricts the generalizability of our findings to male populations, in whom hormonal and physiological differences may affect treatment outcomes; they may require a separate study. Third, our study population consisted exclusively of individuals with Fitzpatrick skin types II and III, which limits the applicability of our results to patients with darker skin types, where safety and efficacy profiles of alexandrite lasers may differ. Fourth, although we used the HowMany AI–based hair counting tool, which demonstrated consistency with manual counts, the possibility of measurement bias or inaccuracy cannot be entirely excluded. Finally, no formal a priori or post hoc power analysis was conducted, and this should be considered when interpreting the findings. Future research with larger, more diverse cohorts, including both sexes and a wider range of skin types, is warranted. Incorporating multivariate models with additional covariates such as age, baseline hair density, and hair thickness may further improve adjustment for potential confounders and enhance the generalizability of results.

Although there are some limitations to the study, it also has significant strengths. In particular, the inclusion of a 12-month follow-up period provides strong evidence of the sustainability and validity of treatment outcomes, which is often lacking in comparative studies. Additionally, reporting detailed treatment parameters enhances the reproducibility of the results and offers practical guidance for clinicians seeking to optimize their alexandrite LHR protocols.

### Conclusions

This study compared single- and dual-flashlamp laser systems for hair removal at a wavelength of 755 nm in individuals with Fitzpatrick skin types II and III. Findings indicated that the method of energy delivery, shaped by flashlamp configuration, rather than the total energy output, significantly influenced treatment efficacy, with both systems achieving comparable hair reduction. While dual-flashlamp systems may enhance device longevity and performance consistency and reduce session time, single-flashlamp systems offer greater cost-effectiveness and are associated with fewer side effects. Further research with larger sample sizes is warranted to refine treatment guidelines and optimize outcomes in LHR.

## Abbreviations

ANCOVA: analysis of covariance
LHR: laser hair removal
